# Process Optimization for Manufacturing Functional Nanosurfaces by Roll-to-Roll Nanoimprint Lithography

**DOI:** 10.3390/nano12030480

**Published:** 2022-01-29

**Authors:** Usama Tahir, Jin Il Kim, Shama Javeed, Amin Khaliq, Jun-Hyun Kim, Doo-In Kim, Myung Yung Jeong

**Affiliations:** 1 Department of Cogno-Mechatronics Engineering, Pusan National University, Busan 46241, Korea; usama@pusan.ac.kr (U.T.); aaminkhaliq@pusan.ac.kr (A.K.); oaksa03@pusan.ac.kr (J.-H.K.); 2 Department of Opto-Mechatronics Engineering, Pusan National University, Busan 46241, Korea; real_one@pusan.ac.kr (J.I.K.); dooin.kim@pusan.ac.kr (D.-I.K.); 3 Department of Mathematics, Pusan National University, Busan 46241, Korea; shamajaveed@pusan.ac.kr

**Keywords:** nanopillars/pores, roll-to-roll imprinting, multiphase flow, imprinting-speed, functional surfaces, sliding mesh method

## Abstract

Roll-to-roll nanoimprint lithography (RTR-NIL) is a low-cost and continuous fabrication process for large-area functional films. However, the partial ultraviolet (UV) resin filling obstructs the ongoing production process. This study incorporates UV resin filling process into the nanopillars and nanopores by using RTR-NIL. A multiphase numerical model with a sliding mesh method is proposed in this study to show the actual phenomena of imprint mold rotation and feeding of UV resin on the polyethylene terephthalate (PET) substrate. The implementation of UV resin filling under environmental conditions was performed by utilizing the open-channel (OC) boundary conditions. The numerical model was solved by using the explicit volume of fluid (VOF) scheme to compute the filling on each node of the computational domain. The effects of different processing parameters were investigated through the proposed numerical model such as imprinting speed (IS), contact angles (CAs), viscosity, initial thickness of the PET, and supporting roll diameter. A good agreement was found between numerical simulations and experimental results. The proposed numerical model gives better insights of the filling process for the mass production of functional surfaces with nanopillars and nanopores patterns for different applications on an industrial scale.

## 1. Introduction

Mass production of nanopatterns by using roll-to-roll UV nanoimprint lithography (RTR-UV-NIL) drew the attention of several researchers because of its practical significance in the micro/nanofabrication industry [1]. The smooth fabrication process has various defects because of the incomplete filling of the UV resin into the nanopatterns from the left/right (based on the filling direction) end of the imprint mold. Several studies [2,3,4,5,6,7,8,9] were conducted in the literature to optimize the filling processes. Pietarinen et al. [10] introduced the micro-optics replication process with the solvent-assisted filling into the micro-pyramid structures to avoid the defects due to air entrapment. Hiroshi et al. [11] used pentafluoropropane gas to replace air during the UV imprinting process. But these methods are difficult to implement on large scale due to experimental limitations. The UV resin filling process in RTR-UV-NIL is hardly explored by the scientific community due to system complexity.

The mathematical models are the most thorough techniques to investigate the RTR-UV-NIL process. Numerous studies [2,3,5,6,7,8,9,12] analyzed the RTR system by using numerical modeling for the UV resin filling process and proposed various strategies to attain high fidelity for the continuous fabrication process. Song et al. [13] investigated the influence of backing film together with UV resin flow into the cavities by utilizing coupled Eulerian–Lagrangian technique. They performed numerical simulations without accounting for air in the model. On an industrial scale, the role of air in RTR-UV-NIL cannot be overlooked. Peng et al. [12] employed multiphase model and investigated the UV resin filling into the pyramid shape cavity by accounting air in their numerical model. Zhou et al. [9] employed multiphase modeling technique and discussed UV resin accumulation as an important parameters during the filling process. A two-body system with an interface between the imprint mold and the outside region was used in their numerical model. In our previous work [5], we utilized multiphase numerical modeling with open-channel (OC) boundary conditions to analyze the filling behavior into the microcavities. The numerical model is based upon a single zone and optimize the filling behavior on the prior and succeeding ends of the imprint mold. However, the numerical modeling of the UV resin filling process into the nanocavities for mass production was not discussed in the literature until now.

In this study, we proposed a theoretical model to optimize the filling of UV resin into the nanocavities for the mass production of functional films. Experiments were carried out utilizing the optimized parameters, resulting in successfully imprinted nanopillars and nanopores patterns without defects. The numerical formulation of the sliding mesh method, geometric model, and explicit finite volume method for the solution to the multiphase problem are presented in Section 2. The numerical simulations’ results are discussed in Section 3. The fabrication of soft molds, roll-to-roll experimental setup, and experimental results based upon numerical simulations are presented in Section 4. Finally, Section 5 concludes this study.

## 2. Numerical Model

**Figure 1:**
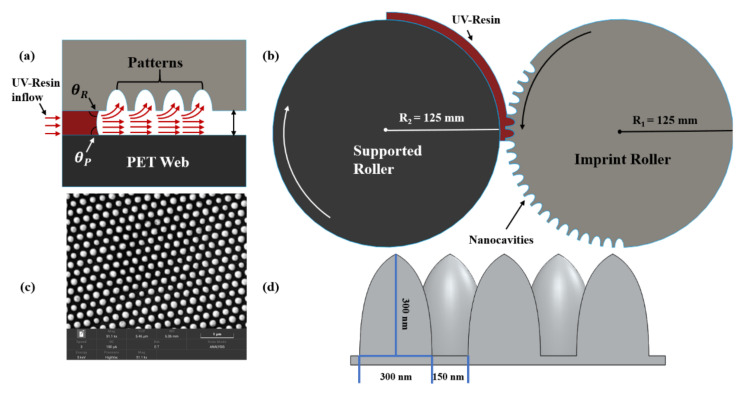
Schematic diagram of ultraviolet (UV) resin filling for elliptical nanocavities. (**a**) Between two parallel plates; (**b**) mass fabrication of nanocavities; (**c**) soft-mold; (**d**) 3D view of nanocavities.

The RTR-UV imprinting technique involves the rotation of the imprint mold, as well as the translational movement of the polyethylene terephthalate (PET) substrate, can be calculated by u=rθ. Here, u and θ represents the translational and rotation speed of PET-Web and imprinting mold. r is the radius of imprint mold and supporting roller. A thoroughly constructed model that includes the entire physics of the problem is required to examine the filling process and optimization of the imprinting speed (IS). The majority of the above studies utilized different numerical models but their models have several limitations, such as the flow of UV resin between two parallel plates and the modeling of a tiny part of the imprinting process without taking air into account, as illustrated in Figure 1a.

To simulate UV resin flow into nanopillars and nanopores under environmental conditions, we used a 2D multiphase volume of fluid model (VOF) model [14,15] with moving walls and OC boundary conditions. The mathematical formulation of multiphase VOF model is explained in [5]. The fixed Eulerian mesh method was used to define the two phases in multiphase VOF model. Since the flow fields of the two phases have the same velocity, a single momentum equation is therefore needed to solve throughout the computational domain in VOF model. Furthermore, according to the specification of current VOF model, the phases cannot overlap or interpenetrate, and their volume fraction is specified between 0 and 1. In addition to the previous study, we employed the sliding mesh algorithm for the rotation of imprint mold. The sliding mesh algorithm is a robust approach for simulating the rotating flows or dynamic motion of one part to another, for example, rotation of blade in centrifugal pump [16]. Although its computational cost is high, it correctly describes the transient behavior of the flow in any problem. In this technique, each cell zone slide is relative to the other cell zone and is connected through an interface between them. The interfaces between two cell zones should be of same shape to employ sliding mesh method accurately; for example, if we slide one cell zone of different types of micro/nanopatterns (V-shape, oval, rectangular) into some area of difference between them, relative to other cell zones, the common interface will give some leeway due to tolerance and a diverging solution to the problem. More precisely we will be unable to solve the problem explicitly in which we kept the Courant–Friedrichs–Lewy (CFL) < 1 to compute the flow at each node of the computational domain. By using backward difference scheme, the general form of fluid transport equation for dynamic sliding mesh for any arbitrary volume U can be written as,

(1) $$\frac{d}{dt}\int_{U}\rho \phi dU=\frac{{\left(\rho \phi U\right)}^{n+1}-{\left(\rho \phi U\right)}^{n}}{\Delta t}$$

Here, $\frac{dU}{dt}$ represents the control volume derivative. The conservation equation to satisfy the mesh can be written as,

(2) $$\frac{dU}{dt}=\int_{dU}{\vec{u}}_{g}.d\vec{A}=\sum_{j}^{n_{f}}{\vec{u}}_{g,j}.{\vec{A}}_{j}$$

where $n_{f}$, ${\vec{A}}_{j}$, and ${\vec{u}}_{g,j}.{\vec{A}}_{j}$ represent the total number faces of control volume, face area vector, and dot product of each control volume, respectively. Equations (1) and (2) update the flow on each node of mesh of the sliding zone with respect to time. The numerical simulations are carried out using the computationally fluid dynamic software ANSYS-Fluent [17] to provide a full overview of the UV filling process into nanopillars and nanopores.

### 2.1. Geometric Model and Open-Channel Boundary Conditions

**Figure 2:**
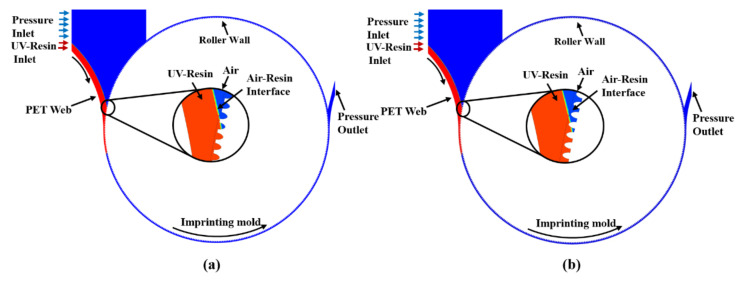
Computational multiphase model scheme with sliding mesh method based upon stationary and fixed zones with open-channel (OC) boundary conditions for (**a**) nanopillars and (**b**) nanopores patterns.

A 2D model was built to explore the filling behavior of elliptical nanopillars and nanopores. Figure 1b depicts the RTR-UV-NIL schematic diagram, which incorporates the movement of the PET-Web from left to right with the assistance of a supporting roller, as well as the rotation of the imprint mold. To assure mechanical precision, the angular velocity of rollers is set in the same way as the linear velocity of the PET-Web. Figure 2a,b shows the detailed simulation model of elliptical nanopillars and nanopores.

UV resin above the PET-Web is driven towards the imprint roller from the left (inlet) side. Above the supporting roller, the PET-Web is treated as a stretched and moving wall boundary condition. The computational domain based on sliding mesh algorithm slides the rotating zone relative to the fixed zone. The rotating zone consist of nanopillars and nanopores and a mesh interface. This mesh interface is connected to the fixed zone interface of same shape, as shown in the magnified view of Figure 2. More precisely, these two cell zones are geometrically separated but numerically connected. The fixed cell zone consists of other parts and boundary conditions of computational domain, such as UV resin supply and OC boundary conditions. The OC-VOF model’s computational domain also includes the initial thickness and velocity of UV resin on the PET substrate. Further, to investigate the UV resin filling process under environmental circumstances, the inflow, outflow, and pressure conditions are set as atmospheric above the PET-Web and imprint roller. The movement of fixed and rotating zones are updated with respect to time in the computational domain.

Table 1 lists the simulation parameters such as height and width of nanopillars/pores, density, viscosity, initial thickness, and CAs for the UV resin filling process. $\theta_{P}$ represents the angle between PET-web and UV resin, and $\theta_{R}$ represents the angle between the imprint roller and UV resin.

### 2.2. Explicit Finite Volume Method

The simulations for both cases (nanopillars and nanopores UV resin filling) were performed using an explicit scheme for finite volume method, because this method is supremum to implicit scheme regarding numerical accuracy when we need to calculate the solution on each node of the computational domain. The transient problem was solved by employing coupled algorithm for pressure–velocity coupling in a pressure-based solver. In spatial discretization, the gradient calculations were performed using the least-squares cell-based method because it provides good accuracy in comparison with that of computationally expensive methods. To reduce the numerical diffusion to solve the momentum equations, a second-order up-wind scheme is utilized by keeping the pressure at PREssure Staggering Option (PRESTO). Further geometric reconstruction scheme was utilized in spatial discretization for face fluxes interpolation.

For temporal discretization, the first-order implicit scheme was used to see the transient behavior of the multiphase problem. The time step was chosen by keeping the Courant number in the acceptable range so that UV resin flow can be computed on each node of the computational domain. The variable time step was set between 2 × 10^−5^ s to 5 × 10^−5^ s to keep the Courant number under 1. The residual during the simulations shows the convergence of the solution up to 10^−6^ s.

A mesh independence study was also implemented for the current study by utilizing the mesh grids of sizes 10 × 10 nm, 25 × 25 nm, and 30 × 30 nm to see the UV resin filling more accurately into the bubble cavity. By employing the above mesh sizes, the difference of bubble elimination into the bubble cavity was less than 1%. It is also investigated by employing the adaptive mesh refinement technique, which also does not affect the filling behavior into the nanocavities. We use four levels of adaptive mesh refinement for each time step during our simulations. The mesh utilized in the simulations is 30 × 30 nm, which was chosen as a compromise between computing time and accuracy [17,18,19].

## 3. Simulation Results

### 3.1. UV Resin Filling Process into the Nanopillars

**Figure 3:**
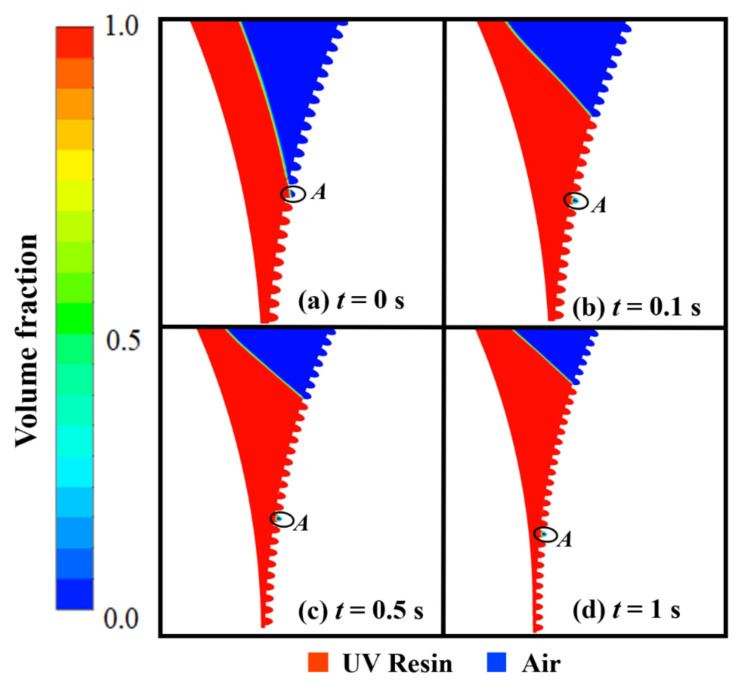
UV resin incomplete filling into elliptical nanopillars with bubble defects: (**a**) *t* = 0 s, (**b**) *t* = 0.1 s, (**c**) *t* = 0.5 s, and (**d**) *t* = 1 s, at a PET-Web speed of 18.75 mm/s with viscosity 200 cp ($\theta_{R}=7.2{}^{\circ}$, $\theta_{P}=16.9{}^{\circ}$).

The filling in elliptical nanopillars at different intervals is shown in Figure 3. Figure 3a depicts the air above the UV resin at time *t* = 0 s. When the imprint mold begins to rotate, the PET-Web delivers UV resin into the nanopillars. The initial thickness of UV resin, viscosity, and PET-Web speed was kept at 1.5 μm, 200 cp, and 18.75 mm/s, respectively. At the same time a little amount of UV resin backflow at the prior end of the imprint roller and successfully fills the nanopillars, except cavity *A* as shown in Figure 3b–d. In cavity *A*, the air is locked above the UV resin and has no way to escape throughout the filling process.

**Figure 4:**
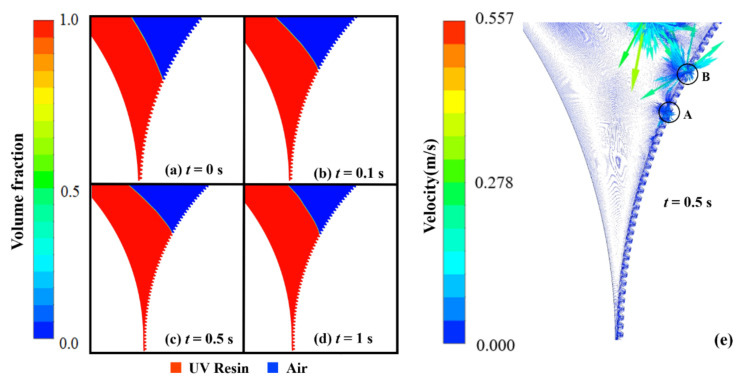
UV resin complete filling into elliptical nanopillars without bubble defects: (**a**) *t* = 0 s, (**b**) *t* = 0.1 s, (**c**) *t* = 0.5 s, and (**d**) *t* = 1 s, at a polyethylene terephthalate (PET)-Web speed of 18.75 mm/s with viscosity 180 cp. (**e**) Velocity vectors of moving PET and rotating roller mold at *t* = 0.5 s.

This kind of bubble entrapment happens due to the contact UV resin initially has with the nanocavities on the imprint mold as shown in Figure 4a. In our numerical model, the imprint mold changes its reference position with time due to the employment of the sliding mesh method, so cavity *A* moves towards the narrow channel where this bubble is impossible to remove during the filling process (see Supplementary Materials Video S1a).

We increase the initial thickness of UV resin above the PET substrate to change the initial contact of UV resin with nanocavities on the imprinting mold (see Supplementary Materials Video S1b). The viscosity and PET-Web speed was kept at 200 cp and 18.75 mm/s during the filling process.

The UV resin successfully fills the cavities at the preceding end of the imprint mold, but a small amount of air is still let into cavity *A*. This problem arises due to the viscosity of UV resin. To resolve this problem, we decrease the viscosity of UV resin to 180 cp during the filling process. Figure 4b–d represents the UV resin filling at different time steps. Nanopillars are successfully filled with UV resin (see Supplementary Materials Video S1c). The UV resin successfully fills the nanopillars at the prior and succeeding ends of the imprint mold during simulations. The processing parameters such as initial thickness, CAs, and PET-Web speed were kept the same as in previous simulation. For the filling process, $\theta_{R}=7.2^{{}^{\circ}}$ and $\theta_{P}=16.2^{{}^{\circ}}$ are the appropriate CAs measured after running several simulations. In numerical simulations, the CA $\theta_{R}$ values vary depending on the pattern shape. As a result, we kept them in a feasible range for successful filling of nanocavities. Figure 4e shows the contours for the velocity vector distribution of UV resin filling and rotational motion of imprinting mold at *t* = 0.5 s.

**Figure 5:**
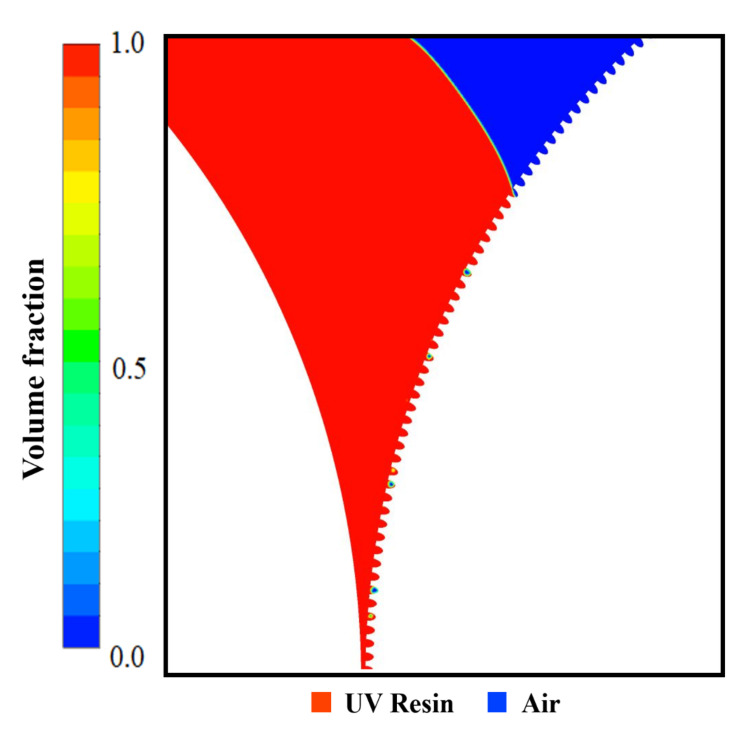
UV resin incomplete filling into elliptical nanopillars with continuous bubble defects at Web speed of 50 mm/s with viscosity 150 cp ($\theta_{R}=7.2{}^{\circ}$, $\theta_{P}=16.9{}^{\circ}$).

**Figure 6:**
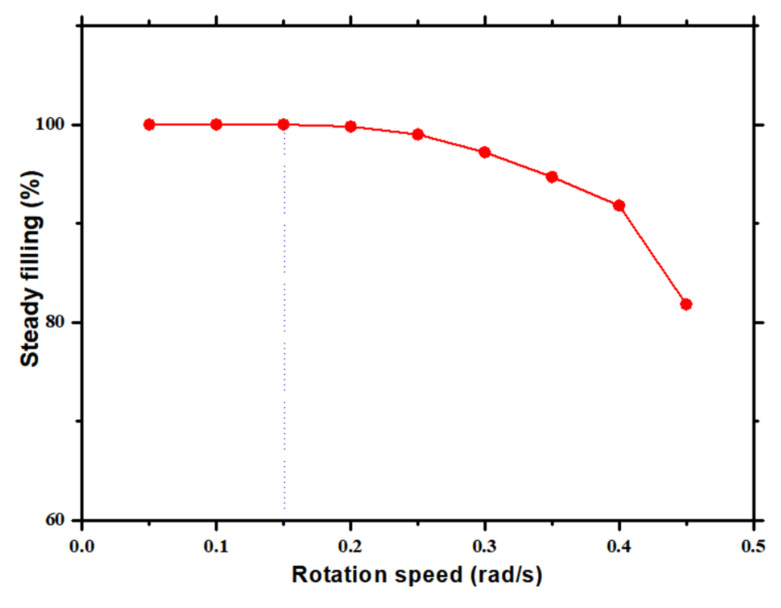
UV resin filling varies from uniform to nonuniform at varying impression mold rotation speeds.

The vector distribution of the velocity of UV resin in cavities *A* and B depicts that UV resin enters the cavity and slows down to form vortices. The velocity distribution of vectors is more in cavity *B* than cavity *A* because the UV resin starts to fill in cavity *B* at *t* = 0.5 s. At high speeds, the imprint mold has less time to fill nanocavities with UV resin. Therefore, bubble entrapment occurs at a high speed. Figure 5 depicts the incomplete filling of UV resin at *t* = 0.5 s with IS at 50 mm/s. Continuous bubble entrapment can be seen in different nanopillars at high IS. Figure 6 shows the UV resin filling process at different ISs. It is also evident that UV resin leaves the nanocavities without filling at high IS.

### 3.2. UV Resin Filling Process into the Nanopores

UV resin filling process into the nanopores is different than the nanopillars due to the reverse filling direction. For simple understanding in the case of hydrophobic surfaces, if the gap between two consecutive nanocavities is less, then fabricated nanosurfaces will show more hydrophobicity for antiwetting phenomena [20]. It is also observed during the numerical simulation that the UV resin easily fills into nanopillars with zero-gap during RTR imprinting, but it is impossible to fill into the nanopores with zero-gap for RTR imprinting because in the reverse direction the filling is impossible between the edges of two consecutive nanocavities.

**Figure 7:**
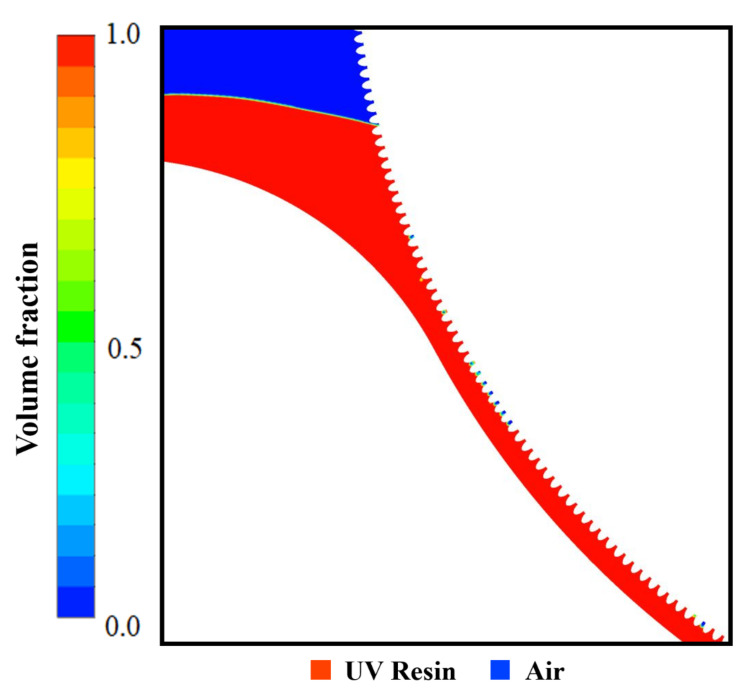
UV resin continuous bubble defects into nanopores with 100 nm gap between edges of two consecutive nanopores at Web speed of 18.75 mm/s with viscosity 180 cp ($\theta_{R}=7.2{}^{\circ}$, $\theta_{P}=16.9{}^{\circ}$).

Therefore, bubble entrapment continuously occurs during the filling process between the consecutive edges of the nanopores. Figure 7 shows the incomplete filling with bubble entrapment between two consecutive nanocavities with a gap of 100 nm at *t* = 0.5 s. We performed numerical simulation by increasing the gap between imprinting mold and supporting roll and also decreasing the supporting roll diameter for the easy escape of air, as shown in Figure 7.

The UV resin fails to fill into the nanocavities because the gap between the edges is very narrow during the filling process. The Ni mold was fabricated based on these investigations and further used in our experiments for mass production of nanopatterns.

Different simulations were performed to optimize the filling process with a suitable gap of 150 nm between two consecutive nanocavities.

**Figure 8:**
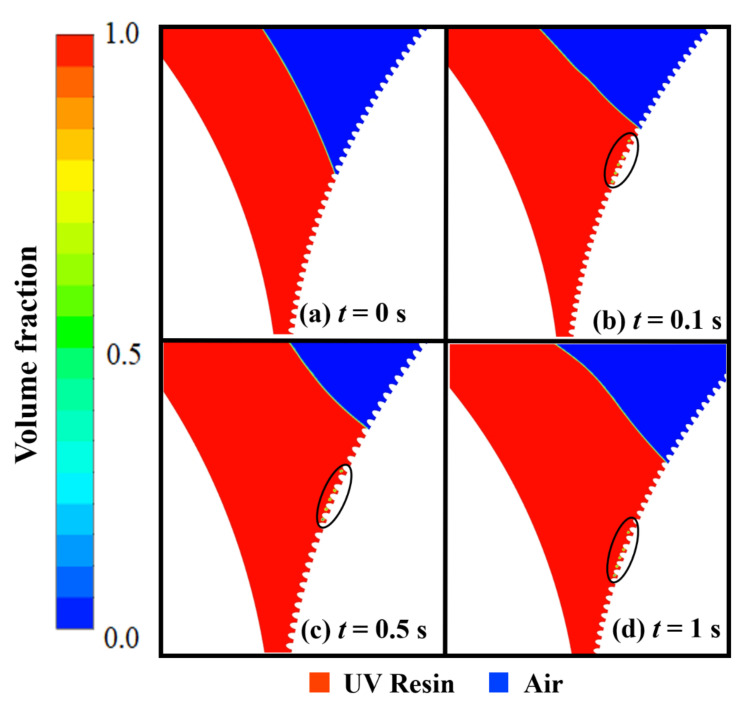
UV resin incomplete filling into nanopores with bubble defects: (**a**) *t* = 0 s, (**b**) *t* = 0.1 s, (**c**) *t* = 0.5 s, and (**d**) *t* = 1 s, at a PET-Web speed of 18.75 mm/s with viscosity 200 cp ($\theta_{R}=7.2{}^{\circ}$, $\theta_{P}=16.9{}^{\circ}$).

**Figure 9:**
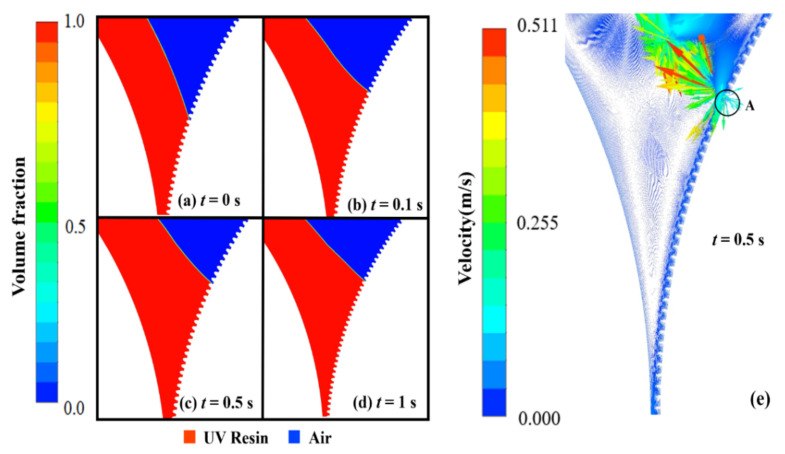
UV resin complete filling into nanopores without bubble defects: (**a**) *t* = 0 s, (**b**) *t* = 0.1 s, (**c**) *t* = 0.5 s, and (**d**) *t* = 1 s, with initial thickness of five micrometer at a PET-Web speed of 18.75 mm/s with viscosity 180 cp. (**e**) Velocity vectors of moving PET and rotating roller mold at *t* = 0.5 s.

Same as nanopillars, bubble entrapment also occurred in the cavities when we kept the UV resin initial thickness of 1.5 μm on the PET substrate (see Supplementary Materials Video S1d). To counter this bubble entrapment, we change the UV resin initial contact with the imprinting mold by increasing the initial thickness of the UV resin above the PET substrate. Figure 8a–d represents the UV resin filling into the nanopores at different time steps. The viscosity and PET-Web speed was kept at 200 cp and 18.75 mm/s during the filling process. Figure 8b–d shows that bubble entrapment significantly decreases by changing the UV resin initial contact with the imprinting mold. However, there are still small bubbles that exist during the filling process due to the viscosity of UV resin (see Supplementary Materials Video S1e). Figure 9 represents the complete filling process of UV resin into nanopores. The UV resin filling process is shown at different time steps in Figure 9a–d.

To get the successful filling at both ends of the imprinting mold without bubble entrapment, we decrease the viscosity of the UV resin up to 180 cp by keeping other parameters the same as in the previous simulation (see Supplementary Materials Video S1f). Figure 9e shows the velocity vectors distribution of UV resin filling and rotation of the imprinting mold at *t* = 0.5 s. Due to surface tension of UV resin and adhesive force from the imprinting mold, the velocity vectors distribution significantly increases in cavity *A* and replaces the air with UV resin.

### 3.3. Effect of Supporting Roller Diameter on UV Resin Filling Process

**Figure 10:**
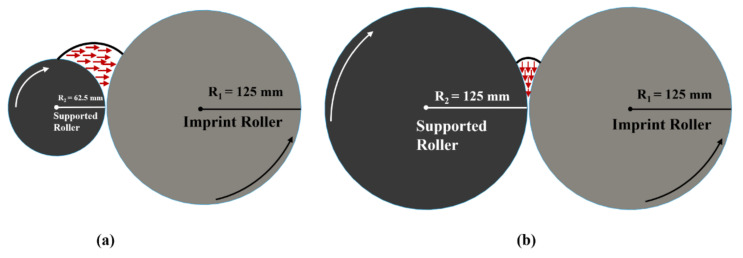
(**a**) Schematic diagram of filling direction of UV resin with supported roller with half of diameter of imprinting mold. (**b**) Schematic diagram of filling direction of UV resin with supported roller and impression mold of same diameter.

In this study, we also investigated the effect of supporting roll diameter during the filling process because it has a direct impact on the initial contact of UV resin with the imprinting mold. The imprint mold and supporting roller of the same diameter are shown in Figure 10a.

**Figure 11:**
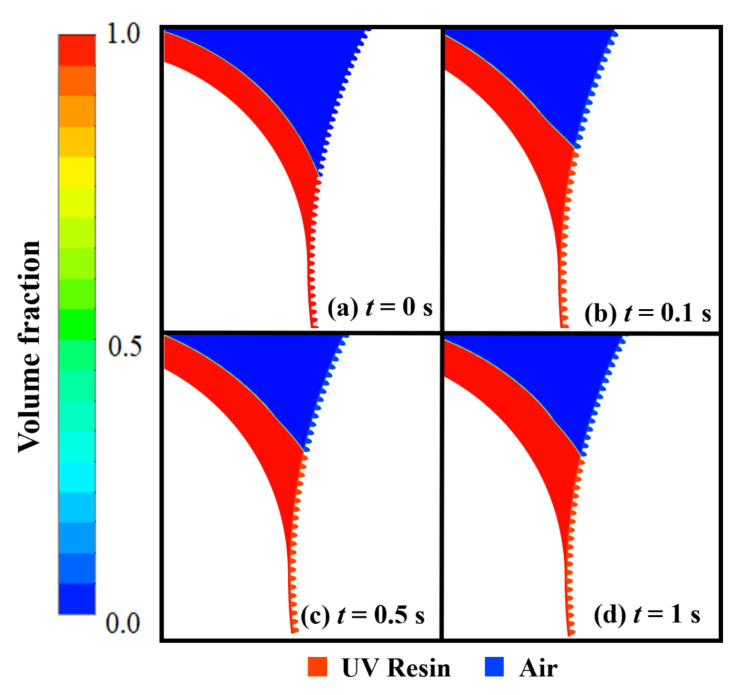
UV resin complete filling into nanopillars without bubble defects with supporting roll diameter of half of imprinting mold: (**a**) *t* = 0 s, (**b**) *t* = 0.1 s, (**c**) *t* = 0.5 s, and (**d**) *t* = 1 s, at a PET-Web speed of 18.75 mm/s with viscosity 180 cp ($\theta_{R}=7.2{}^{\circ}$, $\theta_{P}=16.9{}^{\circ}$).

In this scenario, the UV resin fills vertically into the nanocavities and leaves the bubble entrapment alone if we reduce the initial thickness of UV resin above the PET substrate. If the supporting roll diameter is half of the diameter of the imprinting mold diameter, then UV resin easily goes into the nanocavities during the imprinting process as shown in Figure 10b. Figure 11a–d shows the complete filling of UV resin into the nanocavities at different time steps. Reducing the diameter of supporting roll shows that the UV resin fills the nanocavities with a minimum initial thickness of 1.5 μm on the PET substrate.

## 4. Experimental Section

### 4.1. Fabrication of Nanopillars and Nanopores Soft Molds

The fabrication process of the soft mold with elliptical nanopillars and nanopores patterns consists of three steps: the fabrication of nanopores soft mold from nanopillars nickel (Ni) master mold, surface treatment for smooth release of soft mold, and the fabrication of nanopillar soft mold from treated soft mold. Figure 1c represents the Ni mold or master used in this study, which was obtained by electroplating process [21] that has elliptical nanopillars patterns. The schematic diagram of elliptical patterns with dimensions and the gap between two consecutive nanocavities are shown in Figure 1d. In the first step of the soft mold fabrication process, a uniform coating of 50 μm of UV resin was obtained by using the spin coating method on the Ni master mold. Then, we covered the PET substrate over to well coat UV-curing resin layer and baked it under UV exposure with an intensity of 800 W/cm^2^ for 4 min. The baked UV-curing resin was exposed under the LICHTZEN HG UV lamp system and the liquid state resin transformation to a nontacky-solid state by the photoinitiator. After releasing the PET substate from the Ni mold, the reverse (nanopores) patterns transform onto the PET substrate. In the second step, a surface treatment process was used to reduce the peel-off force during the RTR imprinting process. In this step, the reverse soft mold was dipped into a primer agent for 20 min to develop crosslinking over the patterned surface. The dipped soft mold was kept under room temperature for 1 h to chemically stabilize the cross-linking agent on the surface of the soft mold. Further, the soft mold was again dipped into releasing agent with a fluorinated additive for 20 min to make a low surface energy layer in the soft mold. After forming the low surface energy layer onto the soft mold, it was kept for 24 h under room temperature for chemical stabilization.

This process provides more excellent releasing properties during the imprinting process. In the third step above, the process was repeated again by using negative soft mold for the fabrication of soft mold with positive elliptical nanocavities.

### 4.2. Roll-to-Roll Imprinting Setup

Apart from the imprinting module, the experimental system consists of other modules named as unwinding module, EPC module for PET alignment, slot-die module, dispensing module, demolding module, and rewinding module to support continuous imprinting process. The uncoated PET film is delivered by the unwinding module.

**Figure 12:**
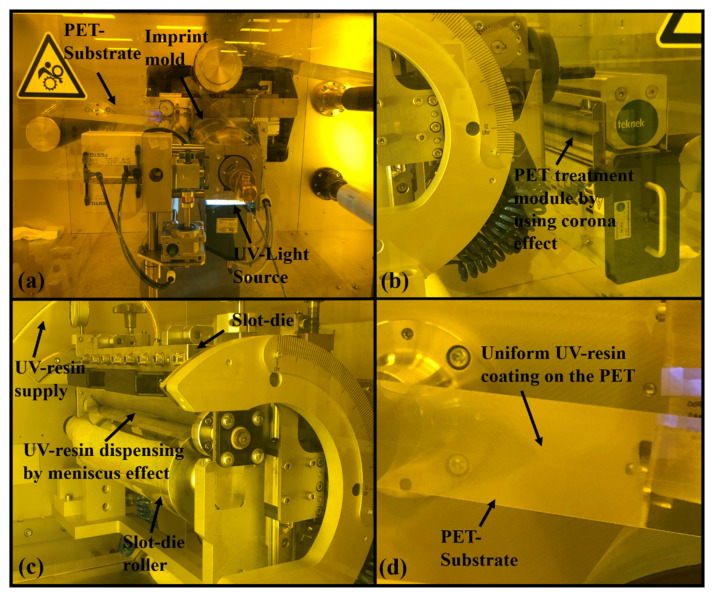
(**a**) Imprinting unit of roll-to-roll (RTR) system with PET-Web of width 250 mm, capable of imprinting the patterns with a speed up to 10 m/min. (**b**) PET substrate treatment unit that incorporates with corona effect to increase adhesion of UV resin with PET substrate. (**c**) Slot-die unit with a dispensing source and dispensing of UV resin on PET substrate by using meniscus effect. (**d**) Uniform coating of height 5 μm of UV resin on PET substrate.

The PET film employed as a substrate was 125 μm thick, with an average transmittance of 87% under visible radiation (Kolon polyester film, Daegu, Korea). The tension module generates tension on the PET film’s ends so that it may correctly align during the imprinting process. The Edge Position Control (EPC) module is responsible for the alignment of PET substrate from unwinding to rewinding module. In this study, we also used a PET substrate treatment module as shown in Figure 12b. This treatment module uses the corona effect to increase the adhesion of UV resin with PET substrate when the coating is applied on the PET substrate. The new slot-die dispensing module is shown in Figure 12c. This module consists of a UV resin injection reservoir that can contain 30 mL of UV resin. This reservoir supplies the UV resin to slot-die with the help of pipe as shown above of the slot die in Figure 12c. Then, slot-die dispenses the UV resin onto the PET substrate by using the meniscus effect. The gap between the slot-die and the roller underneath the slot-die is responsible for the thickness height of UV resin above the PET substrate. In our experiments, we kept this gap up to 5 μm based on our simulations. Figure 12d represents the uniform thickness of 5 μm of UV resin above the PET substrate. The main module of the RTR system is the imprinting module that consists of a supporting roller and imprint-roller of the same diameter of 250 mm as shown in Figure 12a. The imprint mold contains the elliptical nanopillars and nanopores patterns of height and width of 300 nm (AR = 1). To cure the UV resin after filling a UV light is placed underneath the imprint mold.

After curing, in the demolding module, a roll was used above the imprinting roll to detach the imprinted patterns smoothly. The imprinted pattern was dragged back by the rewinding module. Scanning electron microscopy ((SEM) S8000G, TESCAN ORSAY HOLDING, a.s. Brno, Kohoutovice, Brno, Czech Republic) with an electron energy of 5 kV was used to examine the patterns. A layer of Au (40 mA, 4 min) was sputtered on the samples to increase the conductivity for better resolution of SEM images. The UV lamp (UV-LED, UV LED-SPOT-100-HP-IC-365, Dr Hönle AG, Lochhamer Schlag 1 D-82166 Gräfelfing, Munich, Germany) emits the UV light with 1500 mW/cm^2^ intensity on the focus point and cured the UV resin at a wavelength of 460 ± 5 nm. Minuta Technology Co., Ltd. (MINS-311R, Osan, Korea; viscosity: 200 cp at 25 °C) provided the UV resin used in this research.

### 4.3. Experimental Results

In our experimental system, we used a treatment module with a corona effect and slot-die module for the uniform coating of UV resin above the PET substrate. These two modules are quite effective in comparison to coating modules used in previous studies.

**Figure 13:**
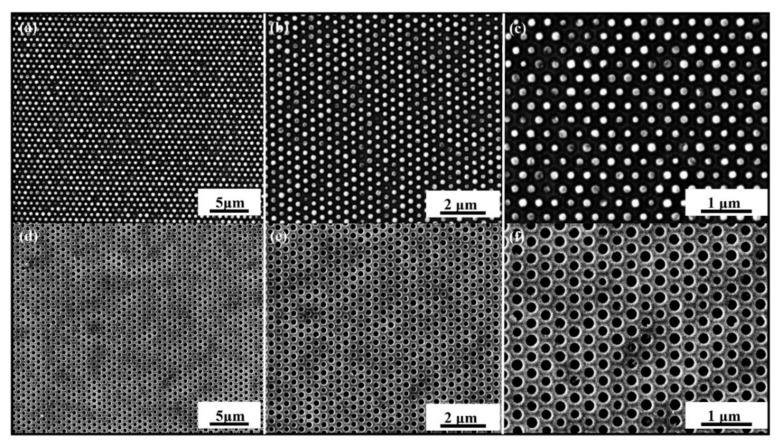
Scanning electron microscopy (SEM) images of fabricated patterns: (**a**) low-magnificent image of elliptical nanopillars via RTR imprinting at Web speed of 18 mm/s; (**b**,**c**) closeup image of nanopillars; (**d**) low-magnificent images of successful imprinting of nanopores; (**e**,**f**) closeup images of nanopores without defects with smooth edges at Web speed of 18 mm/s.

The experiments were performed by utilizing the optimized parameters through the numerical simulations such as viscosity, IS, gap between two consecutive nanocavities, and initial thickness of UV resin above the PET substrate. Figure 13a shows the hexagonal arrangement of elliptical nanopillars. Figure 13b,c represents the top and enlarged view of fabricated nanopatterns by using soft mold through the RTR imprinting process. The diameter, depth, and width of these patterns were 300 nm. Figure 13d shows the SEM images of hexagonal elliptical nanopores of depth 300 nm. Figure 13e,f shows the enlarged view of elliptical nanopores. These SEM images represent the successful imprinting of nanopatterns by using RTR imprinting.

**Figure 14:**
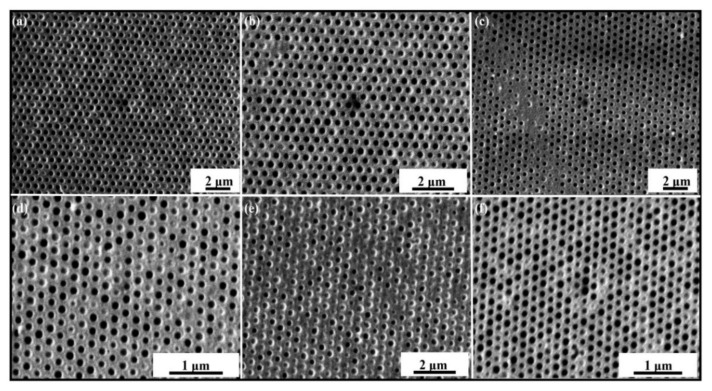
SEM images of fabricated elliptical nanopores with broken edges and defects of incomplete filling at higher IS. (**a**) Low-magnificent image at 30 mm/s; (**b**) closeup image at 30 mm/s; (**c**) 60 mm/s; (**d**) 100 mm/s; (**e**) 130 mm/s, and with maximum speed of the system; and (**f**) 160 mm/s, respectively.

The parameters during the imprinting were kept the same as in the numerical simulations as shown in Figure 4 and Figure 9, respectively. The Web speed for the fabrication of elliptical nanopillars and nanopores was kept 18 mm/min during the experiments. Figure 14a,b shows the top and enlarged view of SEM images for the fabrication of nanopores at an IS of 30 mm/s. At this IS, the fabrication shows the incomplete filling and the edges of nanopores are broken, leading to defects in the quality of the final product. Figure 14c–f shows the SEM images of nanosurfaces at IS 60 mm/s, 100 mm/s, 130 mm/s, and 160 mm/s, respectively. These SEM images show the severe defects and bad arrangement of fabricated patterns with broken edges at high IS. We also carried out experiments on RTR system with different types of nanosurfaces on different IS and found that the suitable speed for RTR nanoimprinting is in the range of 10–20 mm/s.

## 5. Conclusions

The roll-to-roll nanoimprint lithography (RTR-NIL) offers a better solution for the fabrication of nanosurfaces because it is a high-speed and high-resolution manufacturing process. The following findings were reached in this study:

- A multiphase numerical model with open-channel (OC) boundary conditions was utilized in this study together with the sliding mesh method technique. The numerical model optimizes the ultraviolet (UV) resin filling into the nanopillars/pores. The explicit scheme was utilized to calculate the solution on each node of the computational domain. The processing parameters such as IS, viscosity effects, initial thickness on PET substrate, and the effects of supporting roll diameter on filling behavior were optimized by using the proposed numerical model. We also investigated the filling defects at high imprinting speed (IS), and the gap between two consecutive nanopores for UV resin filling was also optimized in this study. The optimal Web speed for the complete filling was noted at 18.75 mm/min. The proposed numerical model is more accurate than the existing models and applicable to any system with variable dimensions of the imprinting molds, and filling behavior with different pattern shapes can also be investigated through this numerical model, such as V-shape, square shape, and nano-lenses shape.
- The 3D hexagonal arrangement of elliptical nanopillar and nanopore soft molds were fabricated by using the soft lithography technique, and these flexible arrays were directly obtained by using the RTR imprinting process under the optimized parameters by using numerical modeling. This is an economical approach that provides a solution for the low-cost fabrication of nanosurfaces. It was also found in the experiments that at high imprinting speed, the fabrication process of nanosurfaces shows continuous defects and broken edges. It was also determined that highly ordered soft molds could be prepared with the established method. In addition, we tested the reproducibility of these soft mold fabrication techniques by running the RTR-NIL process for up to 50 roll revolutions (785.4 m) with a Web speed of 18 mm/s. In summary, these results provide better significance for the economical and continuous mass production of nanosurfaces without defects.

## Figures and Tables

**Table 1:** Processing parameters for numerical simulations.

Parameters	Values
Nanopillars/pores height(H)	300 nm
Nanopillars/pores width (W)	300 nm
Density of UV resin	1196 kg/m^3^
Viscosity of UV resin	150–200 cp
CA $\sigma_{R}$	7.2°
CA $\sigma_{P}$	16.9°
PET-Web Speed	10–50 mm/s
UV resin initial thickness	1–5 micrometer

## Supplementary Materials

The following are available online at www.mdpi.com/article/10.3390/nano12030480/s1, Video S1: UV resin filling in to the nanopillars and nanopores.
